# Effects of Crocin Supplementation during *In Vitro* Maturation of Mouse Oocytes on Glutathione Synthesis and Cytoplasmic Maturation

**DOI:** 10.22074/ijfs.2016.4769

**Published:** 2016-04-05

**Authors:** Elham Mokhber Maleki, Hussein Eimani, Mohammad Reza Bigdeli, Afsane Golkar Narenji, Reyhane Abedi

**Affiliations:** 1 Department of Embryology, Reproductive Biomedicine Research Center, Royan Institute for Reproductive Biomedicine, ACECR, Tehran, Iran; 2 Department of Embryology, Faculty of Biological Sciences, Shahid Beheshti University, Tehran, Iran; 3 Department of Anatomy, Faculty of Medicine, Baqiatallah (a.s.) University of Medical Sciences, Tehran, Iran

**Keywords:** *In Vitro* Maturation, Crocin, Glutathione, Mouse, Oocyte

## Abstract

**Background:**

Crocin is an active ingredient of saffron (*Crocus sativus L.*) and its antioxidant properties have been previously investigated. This carotenoid scavenges free radicals and stimulates glutathione (GSH) synthesis; consequently, it may protect cells against oxidative stress. The aim of this research is to protect oocytes from oxidative stress by the addition of a natural source antioxidant.

**Materials and Methods:**

In the present *in vitro* experimental study, we collected cumulus oocyte complexes (COCs) from mouse ovaries of euthanized, 6-8 week-old female Naval Medical Research Institute (NMRI) mice. Oocytes were subjected to *in vitro* maturation (IVM) in the presence of either crocin (5 or 10 μg/ml), 5 mM buthionine-[S-R]- sulfoximine (BSO), or the combination of crocin plus BSO. Oocytes that matured *in vitro* in a medium without crocin or BSO supplements were considered as controls. Following 16-18 hours of IVM, matured oocytes (n=631) were fertilized by capacitated sperm from NMRI male mice, and cultured *in vitro* for up to 96 hours to assess preimplantation embryonic development. The levels of GSH in metaphase II (MII) oocytes after IVM (n=240) were also assessed by the 5, 5-dithio-bis (2-nitrobenzoic acid) (DTNB)-GSH reductase recycling assay.

**Results:**

Supplementation of IVM media with 10 µg/ml crocin significantly (P<0.05) increased nuclear maturation, preimplantation development and GSH concentrations compared with the control group. Maturation of oocytes in IVM medium supplemented with BSO alone or the combination of 5 µg/ml crocin and BSO drastically decreased GSH concentrations and subsequently resulted in low rates of maturation, fertilization and blastocyst development. However, the combination of 10 µg/ml crocin with 5 mM BSO increased the level of nuclear maturation which was comparable to the control group.

**Conclusion:**

Supplementation of IVM media with crocin can improve nuclear maturation rates and subsequent developmental potential of mouse oocytes. This may occur by its beneficial effect in increasing GSH concentrations in MII oocytes.

## Introduction

Oocyte maturation has two phases: nuclear (visualized by the extrusion of the second polar body) and cytoplasmic (1). Successful maturation, fertilization and development prior to implantation depend on proper growth and differentiation of immature oocytes and the surrounding cumulus cells. In vitro culturing conditions have higher concentrations of O_2_ than in vivo conditions. Oxygen tension in the oviduct is approximately one-quarter to one-third of atmospheric tension (2). This high concentration of O_2_ may cause oxidative stress. Herein, oxidative stress is mediated by reactive oxygen species (ROS) and results in an imbalance of the intracellular redox potential. During *in vitro* maturation (IVM), the oocyte is much more vulnerable to oxidative stress due to ROS overproduction and lack of an innate antioxidant defense system (3, 4). IVM of oocytes and subsequent early embryo development have been shown to be low in the absence of certain supplements such as amino acids and/or antioxidants (5, 6). Recently, numerous attempts have been made to improve the quality and subsequent development of *in vitro* matured oocytes in several species. Modifications to the oocyte culture conditions are considered potential approaches that can help to achieve this improvement. Several studies have shown that addition of antioxidants such as β-mercaptoethanol and vitamins C and E to the culture media could protect the oocytes against oxidative stress that results from IVM (3). 

Throughout history, saffron (*Crocus sativus L.*) has been used as a medicinal plant and a culinary spice (7,8). Among the constituents of saffron stigmas, crocins and crocetin are the most pivotal carotenoids. Investigations into their specific pharmacological properties have led to the discoveries of their antioxidant and antitumor effects (8,11). The crocins are a group of hydrophilic carotenoids that are either monoor di-glycosyl polyene esters of crocetin inside which D-glucose and/or D-gentiobiose occur as carbohydrate residues (12). These carotenoids scavenge free radicals, particularly superoxide anions and, as a result, they may protect cells against oxidative stress (13). According to reports, the methanolic solution of crocin extracted from *Crocus sativus L.* possesses high radical scavenging activity (14,15). Numerous studies have reported anti-apoptotic (16), antiinflammatory (17), and anti-hypertensive (18) therapeutic effects of crocin. In vitro studies show that crocin prevents cell death attributed to oxidative stress (19). 

The aim of the present study was to evaluate the effects of crocin supplementation during IVM of mouse oocytes on nuclear maturation rates, fertilization events, and subsequent preimplantation development after *in vitro* fertilization (IVF). In addition, we assessed glutathione (GSH) concentrations in MII oocytes after IVM in relation to crocin treatment. 

## Materials and Methods

In the present *in vitro* experimental study, all chemicals were purchased from Sigma-Aldrich (Germany) except GSH assay chemicals that were prepared from Wako-Japan. 

## Animals

All procedures performed on animals received the prior approval of the Ethics Board at Royan Institute. Female Naval Medical Research Institute (NMRI) mice, 6 to 8 weeks old and males, 6 to 8 weeks old (Pasteur Institute, Iran), were used in the current study. The animals were kept on a 12 hour light/12 hour dark schedule at an adjusted temperature (20-25°C) and humidity of 50% with ad libitum access to both food and water. Male and female mice were euthanized by cervical dislocation to collect sperm and oocytes. 

## Collection of cumulus oocyte complexes and in vitro maturation

Ovaries (from 70 intact mice) were dissected and transferred into dissecting medium that contained minimum essential medium (α-MEM, Gibco, UK) supplemented with 5% fetal bovine serum (FBS, Gibco, UK), 100 IU penicillin and 100 IU streptomycin for collection of cumulus oocyte complexes (COCs). COCs were retrieved by puncturing antral follicles under a stereomicroscope (Olympus, America) using a pair of 27 gauge needles. IVM was carried out according to Behbahanian et al. (20). Groups of 10-15 COCs were transferred to 30 μl droplets of maturation medium (covered with mineral oil) that consisted of α-MEM supplemented with 100 IU penicillin, 100 IU streptomycin, 5% FBS, 100 mIU/ml recombinant human follicular stimulating hormone (rhFSH, Organon, Holland) and 7.5 IU/ml human chorionic gonadotrophin (hCG, Organon, Holland), then covered with mineral oil. After the incubation of oocytes for 16-18 hours at 37ºC and 5% CO ^2,^some of oocytes were denuded in α-MEM medium supplemented with 40 IU hyaluronidase for the GSH assay. We recorded the percentages of oocytes at the germinal vesicle (GV) stage, the GV breakdown (GVBD) stage (when the GV was absent), and the metaphase II (MII) stage (when the first polar body was extruded) by using an inverted microscope (Nikon, Japan). Other MII oocytes surrounded by cumulus cells were used for IVF. 

## Treatment of oocytes with crocin and/or 5 mM buthionine-[S-R]-sulfoximine

We performed the following treatments to evaluate the effect of crocin and 5 mM buthionine-[S-R]-sulfoximine (BSO) supplementation during IVM on the quality and subsequent development of mouse oocytes. Crocin was dissolved in α-MEM medium and added to the IVM medium at final concentrations of 5 μg/ ml or 10 μg/ml. BSO was dissolved in α-MEM medium and added to the maturation medium either alone at a final concentration of 5 mM or in combination with crocin. 

## Glutathione assay

We evaluated the total intra-cellular GSH concentration in the oocytes by the 5,5-dithiobis(2-nitrobenzoic acid) (DTNB)-GSH reductase recycling assay according to the method described by Viet Linh et al. (21). After IVM and removal of cumulus cells, oocytes were washed four times in phosphate-buffered saline (PBS) that consisted of 0.2 M sodium phosphate buffer and 10 mM EDTA, at pH=7.2. We transferred 10 MII oocytes in to 5 µL PBS in to the bottom of an Eppendorf tube after which 5 µL of 1.25 M phosphoric acid was added to each tube. The tubes were kept frozen at -80°C until the assay was performed. For the assay, oocytes were thawed at room temperature. Then, 175 µl of 0.33 mg/ml nicotinamide adenine dinucleotide phosphate (NADPH) and 25 µl of 6 mM DTNB were added to the tubes. Subsequently, 40 µl of distilled water was mixed within a microfuge tube. Up to 5 µl of 125 U/ml GSH reductase was prepared on ice and added to initiate the reaction. Absorbance was monitored at 412 nm using a spectrophotometer (Ziess, Japan) for 7 times at 30 second intervals (from 0 to 3 minutes). The quantity was determined from a constructed standard curve. For each group and replicate, the experiments were repeated 4 times with 10 MII oocytes. 

## In vitro fertilization

Here and for IVF, we collected the epididymal sperm from the epididymides of male NMRI mice (6-8 weeks old). Spermatozoa were incubated in T6 medium for capacitation. IVF and capacitating medium consisted of T6 medium supplemented with 15 mg/ml bovine serum albumin (BSA, combination of them were equilibrated at 37ºC in 5% CO_2_)(22)Following IVM, MII oocytes surrounded by cumulus cells was washed in IVF media. We transferred 3 or 4 oocytes to 50 μl droplets that were formerly covered by mineral oil. For IVF, 2×10^6^ spermatozoa/ml were added to the droplets that contained the oocytes and the combination of sperm and oocytes were incubated at 37ºC and 5% CO_2_ for 4-6 hours.Then, were corded the numbers of two-pronuclear (2PN) formations observed with an inverted microscope (Nikon, Japan). 

## In vitro embryo culture

Inseminated oocytes were respectively collected, washed, and transferred to 20 μl *in vitro* culture droplets (KSOM with 4 mg/ml BSA) (22). At 72 and 96 hours after IVF, we recorded the numbers Crocin and Cytoplasmic Maturation of Mouse Oocyte of morula and blastocyst embryos with an inverted microscope (Nikon, Japan). 

## Statistical analysis

Both ANOVA and Duncan’s multiple range tests were applied to analyze maturation, 2PN and early development rate by using SPSS 16.0 software. All percentage values were subjected to log transformation prior to analysis. One-way ANOVA and Tukey HSD test were used for the GSH assay. All data were expressed as mean ± SEM. P<0.05 was therefore considered to be statistically significant. 

## Results

### Experiment 1: effects of crocin on *in vitro* maturation, fertilization, and early embryo development

In this experimental study, we cultured the oocytes for 16-18 hours in IVM medium supplemented with either 5 µg/ml or 10 µg/ml of crocin. As depicted in Table 1, compared to the control group, supplementation of the maturation medium with 10 µg/ml crocin significantly increased the percentage of MII oocytes (P<0.05). There were no significant differences in the percentages of GVBD oocytes between treated groups and the control group (P>0.05, Table 1). Addition of 10 µg/ml crocin to maturation medium significantly increased 2PN formation (fertilization rate) compared to the control group (P<0.05, Table 1). Compared to the control group, the oocytes treated with 10 µg/ ml crocin had the highest rate of embryo development and significantly increased blastocyst formation percentages (P<0.05). Significant differences did not exist for the 5 µg/ml dose of crocin in different stages of development (P>0.05). 

### Experiment 2: effects of crocin plus 5 mM buthionine-[S-R]-sulfoximine on *in vitro* maturation, fertilization, and early embryo development 

Oocytes treated with BSO were cultured for 16-18 hours in IVM medium or in medium supplemented with either 5 µg/ml or 10 µg/ml crocin and 5 mM BSO. There was no significant difference between all groups in terms of GVBD percentage. As depicted in Table 2, the percentage of MII oocytes significantly decreased in the BSO treated groups compared to the control group. Compared to BSO alone (P<0.05), the addition of 10 µg/ml crocin to maturation medium with BSO increased the maturation rate to the same level as the control group (P>0.05, Table 2). 

Oocytes matured in the presence of BSO had a significantly lower proportion of 2PN zygotes compared to the control group. Oocytes matured in the presence of 10 µg/ml crocin and BSO exhibited a no significantly higher proportion of 2PN zygotes compared to the groups treated with BSO (P>0.05, Table 2). 

2PN zygotes from each treatment group were cultured further to assess the process of embryo development. The development rate and morula and blastocyst formation in both the BSO treatment and combined treatments of crocin plus BSO greatly declined (P<0.05, Table 2). 

**Table 1:** Percentage of germinal vesicle breakdown (GVBD), metaphase II (MII), two-pronuclear (2PN), morula and blastocysts in crocin treatments


		After 4 hours	After 16-18 hours		After 4-6 hours			72 hours after IVF	96 hours after IVF	
Crocin	Total COC	GVBD (%)	MII	P value(%)	Total MII inseminated	2PN formation	P value(%)	8-cell-morula (%)	Blastocyst	P value (%)

0	97	90 (92 ± 3)^a^	51 (52 ± 7)^a^		51	38 (70 ± 7)^a^		14 (40 ± 12)^a^	9 (20 ± 7)^a^	
5 μg/ml	98	92 (94 ± 2)^a^	65 (65 ± 7)^a^	P=0.18	65	57 (84 ± 4)^a^	P=0.061	20 (43 ± 8)^a^	14 (24 ± 10)^a^	P=0.19
10 μg/ml	104	97 (93 ± 2)^a^	79 (75± 3)^b^	P=0.02	75	67 (90 ± 3)^b^	P=0.013	32 (47 ± 7)^a^	29 (43 ± 3)^b^	P=0.03


Percentage of 2PN, 8-cell or morula, and blastocyst embryos in relation to 2PN cells. Data are expressed as mean ± SEM. All experiments have been repeated seven times. Different superscripts indicate significant differences (P<0.05) and similar superscripts show no significant differences in a column (P>0.05). IVF; In vitro fertilization, COC; Cumulus oocyte complex and P value; Comparison between the experimental and control groups.

**Table 2:** Percentage of germinal vesicle breakdown (GVBD), metaphase II (MII), two-pronuclear (2PN), morula and blastocysts in crocin and 5 mM buthionine-[S-R]-sulfoximine (BSO) treatments


		After 4 hours	After 16-18 hours		After 4-6 hours			72 hours after IVF	96 hours after IVF	
Crocin	Total COC	GVBD (%)	MII	P value(%)	Total MII inseminated	2PN formation	P value(%)	8-cell-morula (%)	Blastocyst	P value

0	0	95	89 (93 ± 5)^a^	52 (55 ± 5)		39 (74 ± 2)^a^		15 (45 ± 7)^a^		7 (20 ± 3)^a^	
0	5 mM	80	65 (85 ± 9)^a^	33 (42 ± 2)^b^	P=0.024	15 (47 ± 6)^b^	P=0.003	0^b^	P=0.000	0^b^	P=0.000
5 μg/ml	5 mM	77	72 (93 ± 3)^a^	33 (43 ± 3)^b^	P=0.034	17 (52 ± 6)^b^	P=0.022	2 (7 ± 7)^bc^	P=0.000	0^b^	P=0.000
10 μg/ml	5 mM	80	75 (93 ± 2)^a^	51(64 ± 4)^a^	P=0.021	29 (59 ± 5)^b^	P=0.042	4 (11 ± 5)^c^ p=0.002		2 (5 ± 3)^b^	P=0.001


Percentage of 2PN, 8-cell or morula, and blastocyst embryos in relation to 2PN cells. Data are expressed as mean ± SEM. All experiments have been repeated seven times. Different superscripts indicate significant differences (P<0.05) and similar superscripts show no significant differences in a column (P>0.05). IVF; In vitro maturation, BSO; Buthionine-[S-R]-sulfoximine, GVBD; Percentage of germinal vesicle breakdown, MII; Metaphase II oocytes, 2PN; Two-pronuclear, COC; Cumulus oocyte complex and P value; Comparison between the experimental and control groups.

### Experiment 3: glutathione content of *in vitro* matured oocytes following maturation in crocin supplemented medium 

Intracellular concentration of GSH among oocytes later to their maturation in culture media supplemented with 5 µg/ml or 10 µg/ml crocin was measured. We assayed four samples (40 oocytes per treatment) from four replications for the purpose of each treatment. Compared to the control group, the GSH concentration increased significantly in the group treated with 10 µg/ml crocin (P<0.05). Addition of 5 µg/ml crocin to the medium did not generate any significant difference compared to the control group (P>0.05, Table 3). 

### Experiment 4: glutathione content of *in vitro* matured oocytes in the presence of crocin and 5 mM buthionine-[S-R]-sulfoximine 

The GSH content of the oocytes matured in the presence of BSO significantly decreased compared to the control group. Compared to the group treated with BSO alone, addition of 5 µg/ml or 10 µg/ml crocin to BSO treated oocytes did not significantly affect GSH content of MII oocytes after maturation (P>0.05, Table 3). 

**Table:** 


After oocyte maturation (16-18 hours)			
Crocin	BSO	Total GSH (pmol/oocyte)	P value

0	0	2.24 ± 0.43^b^	
5 μg/ml	0	2.71 ± 0.58^ab^	p=0.92
10 μg/ml	0	3.83 ± 0.29^a^	p=0.046
0	5 mM	0.27 ± 0.09c	p=0.045
5 μg/ml	5 mM	0.42 ± 0.15^c^	p=0.018
10 μg/ml	5 mM	0.64 ± 0.28^c^	p=0.010


Data are expressed as mean ± SEM. All experiments were repeated four times. Different superscripts indicate significant differences (P<0.05) and similar superscripts show no significant differences in a column (P>0.05). P value; Comparisons between each experimental and the control group.

## Discussion

The presence of high quality oocytes prior to IVF is an important factor that affects developmental competence of subsequent embryos (23). One of the most crucial and harmful factors which can affect *in vitro* oocytes and embryo development are free radicals. Free radicals have deteriorating effects on DNA repair, oocyte maturation, and meiotic spindle assembly (24). This research has investigated the improving effect of crocin (10 µg/ml) added to maturation medium on oocyte maturation, 2PN formation, and developmental competence of oocytes. However the lower dosage of crocin (5 µg/ml) did not affect either the maturation rate or the 2PN and blastocyst formation rates. Former *in vivo* investigations of different organs indicated positive effects of saffron extract and crocin against adverse effects of free radicals and oxidative stress (25,27). *In vitro* researches also demonstrated and emphasized the antioxidant properties of crocin (16,28,30). 

Assimopoulou et al. (14) reported that a metabolic solution of crocin extracted from *Crocus sativus L.* possessed a high level of radical scavenging activity. *In vitro* studies determined the direct linkage between total crocin concentration and antioxidant properties. Antioxidant function has seemed to be strongly influenced by the attached sugar moieties in crocin structures (31,32). This natural antioxidant can impact cells by different mechanisms including nitrite scavenging ability, 2, 2-azino-bis (3-ethylbenzthiazoline-6-sulfonic acid) radical cation inhibition, SOD-like activity, and elongation of lipid peroxidation (6). The radical scavenging potential of crocin is the source of its neuro-protective, anti-aging, anti-inflammatory, and anti-tumor activities (33). An *in vitro* experiment has shown that crocin can modulate cellular proteins and alter their functions. It can particularly affect several cell processes through the interaction with tubulin proteins or microtubules as an important cytoplasmic protein which affects cell division (34). We have observed in the current study, that the higher maturation rate caused by crocin indicated its positive effects during the *in vitro* culture. As observed, higher dosages of crocin (10 µg/ml) increased the oocyte maturation percentage. The effect of crocin appeared to be dose-dependent. By the time the 10 µg/ml dose of crocin was added to the maturation medium, the higher quality of *in vitro* matured oocytes resulted in a higher capacity for 2PN formation and embryonic development efficiency. Although the lower dosage of crocin (5 µg/ml) increased IVM, 2PN and blastocyst formation rate compared to the control group, this finding was not significant. The increased effects of the higher crocin dosage (10 µg/ml) on IVM, 2PN and early embryonic development rate were probably due to its dose-dependent antioxidant effects. 

The current study results of the GSH assay have revealed that the addition of crocin (10 µg/ml) to the maturation medium increased the GSH content of MII oocytes after IVM. GSH is one of the fundamental non-enzymatic defensive structures against ROS in the mammalian oocyte and embryo. GSH is considered an indicator of cytoplasmic maturation in oocytes (35,36). GSH content increases during development and oocyte maturation in the ovary, as the oocyte approaches the time of ovulation, and protects oocytes at later stages of fertilization (37). In oocytes, GSH stabilizes the meiotic spindle against oxidizing agents and is involved in the enhancement of MII, normal formation of the egg, male pronucleus formation, and inhibition of two-cell stage arrest (38). 

After fertilization, GSH participates in sperm decondensation parallel to oocyte activation and transformation of the fertilizing sperm head into the male pronucleus (39). *In vitro* culture conditions exacerbate the formation of ROS, which exert oxidative stress and deplete intracellular GSH content in the oocyte and embryo (40). A decline in oocyte GSH levels results in failure of 2PN formation and reduced embryo development (41). Previous studies have found that crocin can enhance the activities of GSH reductase and a rate-limiting enzyme, γ-glutamyl cysteine synthetase (γ-GCS), which is an enzyme involved in the synthesis of GSH. Thus it can contribute to a stable GSH supply in PC12 cells *in vitro* (42,43). A series of studies by Soeda et al. (44) have indicated that a GSH dependent mechanism is involved in the inhibitory effects of crocin on oxidative stress-induced cell death. There are *in vivo* studies which indicate the effects of crocin on recovering levels of GSH and antioxidant enzymes against oxidative stress (45,46). 

The increased rate of GSH contents in the cytoMokhber Maleki et al. plasm during IVM with the addition of 10 µg/ml crocin suggested that aside from the different antioxidant functions of crocin, increased GSH was one of the main causes of increased pronucleus production and early developmental competence. 

The effect of crocin increased the rate of IVM and subsequent *in vitro* development of oocytes. Addition of BSO caused depletion of GSH in the two groups of lower and higher crocin dosages. Depletion in the concentration of GSH caused by BSO supplementation affected oocyte maturation rate. However, the combination of crocin 10 µg/ml and BSO had no significant effect on maturation compared to the control group. The lower rate of subsequent developmental competence in all BSO treated groups appeared to be related to the lower preservation of GSH which was probably due to the impact of BSO during IVM. BSO decreased GSH production and it appeared that crocin addition did not compensate for this depletion. 

Tavana et al. (47) have reported that higher dosages such as 40 µg/ml of saffron extract in maturation medium increases maturation rate. However, in order to have better IVF and embryo development, addition of lower saffron dosages such as 5 µg/ml is more effective. Our research has indicated that crocin (5 µg/ml) as a component of saffron did not affect any aspects of oocyte and embryo culture. Crocin, at the lower dosage, did not induce GSH synthesis and therefore it had lower antioxidant abilities. Saffron extract has been depicted to have higher antioxidant activities due to the presence of other components with antioxidant properties and synergic effects (48). We have reported that higher dosages of crocin (50, 100, 400 µg/ml) did not have any positive effect on IVF and embryo development. It seems that crocin affects cultured oocytes on a dose-dependent manner; hence 10 µg/ml of crocin would be the best dosage for supplementation. 

## Conclusion

Supplementation by crocin, an active ingredient of *Crocus sativus L.*, during IVM of oocytes can improve maturation, fertilization and early embryo development outcomes. Crocin can dose-dependently increase ooplasmic GSH concentration and cytoplasmic maturation during the maturation process. By the inhibition of GSH synthesis, BSO appears to have an inhibitory effect on crocin efficiency in maturation medium. 
